# Genome-Wide Identification of *WRKY* Genes and Their Responses to Chilling Stress in *Kandelia obovata*

**DOI:** 10.3389/fgene.2022.875316

**Published:** 2022-03-31

**Authors:** Zhaokui Du, Shixian You, Xin Zhao, Lihu Xiong, Junmin Li

**Affiliations:** ^1^ Zhejiang Provincial Key Laboratory of Plant Evolutionary Ecology and Conservation, Taizhou University, Taizhou, China; ^2^ Yuhuan Municipal Bureau of Natural Resources and Planning, Yuhuan, China; ^3^ Marine Academy of Zhejiang Province, Hangzhou, China

**Keywords:** expression profiles, *Kandelia obovata*, low temperature, phylogenetic analysis, WRKY transcriptional factor

## Abstract

**Background:** *Kandelia obovata*, a dominant mangrove species, is widely distributed in tropical and subtropical areas. Low temperature is the major abiotic stress that seriously limits the survival and growth of mangroves. WRKY transcription factors (TFs) play vital roles in responses to biotic and abiotic stresses. However, genome-wide analysis of *WRKY* genes in *K. obovata* and their responses to chilling stress have not been reported.

**Methods:** Bioinformatic analysis was used to identify and characterize the *K. obovata WRKY* (*KoWRKY*) gene family, RNA-seq and qRT–PCR analyses were employed to screen *KoWRKYs* that respond to chilling stress.

**Results:** Sixty-four *KoWRKYs* were identified and they were unevenly distributed across all 18 *K. obovata* chromosomes. Many orthologous *WRKY* gene pairs were identified between *Arabidopsis thaliana* and *K. obovata*, showing high synteny between the two genomes. Segmental duplication events were found to be the major force driving the expansion for the *KoWRKY* gene family. Most of the *KoWRKY* genes contained several kinds of hormone- and stress-responsive *cis*-elements in their promoter. KoWRKY proteins belonged to three groups (I, II, III) according to their conserved WRKY domains and zinc-finger structure. Expression patterns derived from the RNA-seq and qRT–PCR analyses revealed that 9 *KoWRKY*s were significantly upregulated during chilling acclimation in the leaves. KEGG pathway enrichment analysis showed that the target genes of KoWRKYs were significantly involved in 11 pathways, and coexpression network analysis showed that 315 coexpressed pairs (*KoWRKYs* and mRNAs) were positively correlated.

**Conclusion:** Sixty-four *KoWRKYs* from the *K. obovata* genome were identified, 9 of which exhibited chilling stress-induced expression patterns. These genes represent candidates for future functional analysis of *KoWRKYs* involved in chilling stress related signaling pathways in *K. obovata*. Our results provide a basis for further analysis of *KoWRKY* genes to determine their functions and molecular mechanisms in *K. obovata* in response to chilling stress.

## Introduction

Cold stress, including both chilling stress (0–15°C) and freezing stress (<0°C), is a significant abiotic stress to plants (Jiang et al., 2021; Wu et al., 2021). Plants from tropical and subtropical regions usually show sensitivity to cold stress and are even vulnerable to chilling stress due to the inability for cold acclimation (Wu et al., 2019). Mangroves are important components of coastal ecosystems in many tropical and subtropical regions of the world and play key roles in these ecosystems through various ecological, social, and economic functions (Li et al., 2021). However, mangroves are fragile ecosystems that are often threatened by flooding, high salinity, anaerobic soils, and extreme climate events (Giri et al., 2011). Among all of these abiotic stresses, cold stress is considered a vital environmental factor limiting the growth and geographical distribution of mangrove plants (Fei et al., 2015). For example, white mangrove (Laguncularia racemosa) trees in a northern region of Tampa Bay, Florida, were exposed to freezing temperatures (−2°C) for 8 h in January 2003, and the leaves of these trees noticeably withered as a result of this freezing (Ellis et al., 2006). In southern China, a chilling temperatures occurred in early 2008, and a large number of mangrove plants withered and even died (Chen et al., 2017). Research involving remote sensing technology showed that cold spells affected northeastern Asia in January 2021, confirming that the canopies of mangrove stands had been stressed by low temperatures (Peereman et al., 2021).

*Kandelia obovata*, a species in the mangrove family Rhizophoraceae, has been reclassified as a new species that was previously recognized as *Kandelia candel* in regions of China and Japan (Sheue et al., 2003). As a dominant species of mangrove forest in Eastern Asia, *K. obovata* provides critical ecosystem services to the human beings, including coastal protection, habitat provision, biodiversity maintenance, water purification, and carbon sequestration (Wu et al., 2020; Wei et al., 2022). It is well known that the latitudinal distribution of mangroves is mainly limited by temperature. *K. obovata* has relatively high tolerance against low-temperature stress and is a major mangrove species distributed along the southeastern coastline of China (Chen et al., 2017). In China, *K. obovata* is naturally distributed in southern Fuding (27° 20′ N), Fujian Province (Wang et al., 2010). However, climate change has led to the expansion of various mangrove species to higher latitudes (Osland et al., 2013), and *K. obovata* was successfully introduced to Zhoushan (29° 30′ N), Zhejiang Province, in 2016. A successive freezing spell (minimum −3.2°C) occurred in the winter of 2010 on Ximen Island (28° 25′ N), Yueqing, Zhejiang Province, China, and the leaves of *K. obovata* had become brown and wilted, and a high mortality of 2-year-old seedlings was reported (Zheng et al., 2016).

In recent years, many studies have provided valuable insight into the physiological mechanisms (Zheng et al., 2016; Liu et al., 2018; Wang et al., 2019) and molecular mechanisms (Peng et al., 2015; Fei et al., 2021) underlying the response of *K. obovata* to low temperature. In our previous study, we cloned the AP2/EREBP transcription factor (TF) KcCBF3 and demonstrated that it might participate in the adaptation of *K*. *obovata* to low-temperature stress (Du and Li, 2019). TFs can bind to *cis*-elements or interact with other regulatory factors to regulate the expression of downstream defense-related genes (Manna et al., 2021). Increasing numbers of reports show that a number of different TFs, including AP2/EREBP, bHLH, MYB, bZIP, NAC, WRKY, and other TFs, play important regulatory roles in plant stress responses (Gahlaut et al., 2016; Meraj et al., 2020).

WRKY proteins (WRKYs), which constitute the largest family of TFs among all TFs, can identify and bind to W-box [(C/T)TGAC(C/T)] *cis*-elements in the promoter of their target genes; WRKYs are approximately 60 amino acids in length and contain one or two highly conserved heptapeptide WRKYGQK motifs and a typical zinc-finger C_2_H_2_ (CX_4-5_Cx_22-23_HXH) or C_2_HC (CX_7_CX_23_HXC) domain at their C-terminus (Eulgem et al., 2000). In addition to the conserved sequences of the WRKYGQK motifs, some variants, including WRKYGEK, WRKYGRK, WKKYGQK, and WKRYGQK, can also be found in plants (Jiang et al., 2017). Based on the number of WRKY domains and the features of their zinc finger motifs, WRKYs are usually divided into three groups (I, II and III). The group I proteins have two WRKY domains, while the group II proteins, which have only one WRKY domain and a C_2_H_2_ zinc-finger motif, can be further subdivided into five subgroups (i.e., IIa–e) based on their phylogenetic relations. Proteins from group III have one WRKY domain and a C_2_HC motif (Eulgem et al., 2000).

By functioning synergistically with different genes and other TFs, WRKYs play important roles in plants in the defense against pathogens (bacteria, fungi, and viruses) (Jiang et al., 2017). Studies have also indicated that WRKYs are involved in regulating gene expression under abiotic stresses, such as cold, heat, drought and salinity stresses. In addition to roles in response to abiotic and biotic stresses, WRKYs also participate in various plant processes involving germination, growth, development, senescence and metabolic pathways (Rushton et al., 2010). Many studies have demonstrated that members of the WRKY family play essential regulatory roles in the cold stress response. Ten WRKY genes were shown to be strongly induced in *Solanum lycopersicum* during cold stress, and 12 WRKYs were significantly downregulated (Chen et al., 2015). Fifty-nine WRKY genes have been identified in the *Vitis vinifera* genome, and more than ten of them showed stress-induced expression patterns in response to cold (Wang et al., 2014). Kim *et al.* (2016) reported that the rice WRKY TF *OsWRKY71* has a positive function in cold tolerance by regulating downstream target genes. In contrast, the TF WRKY34 negatively mediates the cold sensitivity of mature pollen of *Arabidopsis thaliana* (Zou et al., 2010).

Despite the important role of WRKYs in different species under abiotic stress, there is no information on these proteins in *K. obovata*. Chilling stress represents one of the major environmental stresses that severely affects the distribution and survival of *K. obovata* by negatively affecting the hypocotyl, impairing the photosynthetic apparatus, and stunting growth. Therefore, screening for chilling tolerance genes and improving the cold tolerance of *K. obovata* is particularly important against the background of the current increase in occasional extreme weather events. The aim of the current research was to identify *KoWRKY* genes in the *K. obovata* genome, to classify their expression patterns and to reveal the putative targets and their underlying regulatory biological processes under chilling stress. Our results might provide insight into the molecular importance of *KoWRKY*s under chilling stress.

## Materials and Methods

### Plant Material and Treatments

Healthy and mature propagules of *K. obovata* were collected from Yueqing Bay, Yuhuan city, Zhejiang Province, China (28°13′N, 121°10′ E). The propagules were cultivated in a growth chamber (25°C temperature, 75% humidity, 14 h light/10 h darkness photoperiod) in plastic pots containing sand and watered with 1/2-strength Hoagland’s nutrient solution weekly. At the six-leaf stage, the seedlings were cultivated under chilling stress (4 °C) for 0, 1, 3, and 12 h. All the treatments included three seedlings. The leaves were collected, frozen immediately in liquid nitrogen and then stored at −80 °C.

### Identification of *KoWRKY* Genes From *K. obovata* Genome

The complete genome sequence files of *K. obovata* were downloaded from the Genome Sequence Archive (GSA) webpage (https://bigd.big.ac.cn/gsa/browse/CRA002395). Information on the WRKY domain hidden Markov model (HMM) profile numbered PF03106 was retrieved from the Pfam protein family database (http://pfam.sanger.ac.uk/), and the *A. thaliana* WRKY (AtWRKY) sequence information was obtained from The Arabidopsis Information Resource (TAIR) ( www.arabidopsis.org ). The candidate WRKY protein sequences were identified by comprehensive research using HMMER (E-value cutoff <1E-5) and BLAST analyses (72 AtWRKYs were used as queries) of the *K. obovata* whole-genome and protein databases. These KoWRKY sequences were identified by checking the complete WRKY conserved domain with SMART (http://smart.embl-heidelberg.de/) and InterPro (http://www.ebi.ac.uk/interpro/) and were confirmed manually by checking all sequences containing WRKYGQK motifs and a typical C_2_H_2_ (CX_4-5_Cx_22-23_HXH) or C_2_HC (CX_7_CX_23_HXC) zinc-finger domain. The sequences of the confirmed KoWRKYs were input into the ExPASy online tool website (http://web.expasy.org/protparam/) to calculate the physicochemical properties of the proteins, including the molecular weight (MW) and isoelectric point (pI). The subcellular localization of the KoWRKYs was determined by ProtComp 9.0 (http://linux1.softberry.com/berry.phtml?topic=protcomppl&group=programs&subgroup=proloc).

### Analysis of Chromosomal Localization, Gene Structure and *Cis*-elements in the Promoter Regions of *KoWRKYs*

The chromosomal positions of all the *KoWRKY* genes were determined from the genome annotation file. The physical positions of the *KoWRKY* genes on the chromosome were mapped using MapChart (Voorrips, 2002). The exon/intron structure of the *KoWRKY* genes was determined by comparing their predicted coding sequence (CDS) with genomic sequences using the gene structure display server (http://gsds.cbi.pku.edu.cn/). The upstream 2 kb sequences from the transcription initiation site of all the *KoWRKY* genes were extracted, and the *cis*-elements in these regions were identiﬁed using the PlantCARE database (http://bioinformatics.psb.ugent.be/webtools/plantcare/html/).

### Analysis of Gene Duplication, Selective Pressure, and Collinearity of *KoWRKYs*

The criteria used for identifying duplicate genes were as follows: the similarity of the two aligned sequences was greater than 75%, and the length of the shorter aligned sequence covered more than 75% of that of the longer sequence. Two or more adjacent duplicates on the same chromosome within 100 kb were defined as tandem duplications, while duplicates across different chromosomes or at a distance greater than 100 kb on the same chromosome were considered segmental duplications (Kan et al., 2021). The synonymous rate (Ks) and nonsynonymous rate (Ka) of the identified *KoWRKY* gene pairs were calculated using KaKs Calculator version 2 (Wang Y et al., 2012). The approximate date [million years ago (Mya])] of each duplication event was estimated using the mean Ks values with the formula T = Ks/2λ, in which the mean synonymous substitution rate (λ) was 6.1 × 10^–9^ (Lynch and Conery, 2000; Zhu et al., 2014). OrthoFinder with default parameters was employed to identify homologous genes between two different genomes (*K. obovata* and *A. thaliana*), and a synteny graph was constructed with TBtools according to the identified homologous gene pairs (Chen et al., 2020a).

### Analysis of Motif, Conserved Domain and Phylogenesis of KoWRKYs

Multiple EM for Motif Elicitation (MEME) was used to analyze the KoWRKYs and to identify 15 possible conserved motifs (http://meme.nbcr.net/meme/intro.html). The parameters were as follows: the repetitive time was “any”, the maximum motif number was 15, and the motif width was between 5 and 50 residues. The MEME results were subsequently displayed with TBtools software (Chen et al., 2020a). Multiple sequence alignment of the KoWRKY domains was performed using MAFFT version 7 (Katoh and Standley, 2013). Based on the alignment of the WRKY domains of the KoWRKYs and AtWRKYs, a phylogenetic tree was constructed with FastTree version 2 *via* the generalized time-reversible (GTR) model in conjunction with the Shimodaira–Hasegawa (SH) test (Price et al., 2010). All the identified KoWRKYs were divided into different groups according to the classification of AtWRKY sequences.

### KoWRKY Target Gene Prediction and Kyoto Encyclopedia of Genes and Genomes Analysis

The 2-kb DNA sequences upstream of the ATG start codon of all genes assembled from the *K. obovata* genome were used to identify WRKY TF-binding sites. The potential *KoWRKY* target genes that were predicted with PlantRegMap were used for further pathway enrichment analysis with the KEGG database (https://www.kegg.jp/kegg/pathway.html) (Tian et al., 2019). Hypergeometric Fisher’s exact test (*p* < 0.01) and the Benjamini test [false discovery rate (FDR])<0.05] were performed to detect statistically significantly enriched KEGG pathways. The R package ggplot2 was used to visualize the top 11 significantly enriched KEGG pathways.

### Expression Proﬁles of *KoWRKY* Genes in Different Tissues and in Response to Chilling Stress Based on RNA-Seq

Transcriptomic data of *K. obovata* were obtained from the National Center for Biotechnology Information (NCBI) publicly accessible database (Accession number: PRJNA678025). The expression levels of the *KoWRKY* genes were analyzed in various tissues, including root, stem, leaf, flower, pistil, stamen, sepal, and fruit tissues. The expression data were transformed into log_2_ [transcripts per million (TPM)+1] values for differential expression analysis (Zeng et al., 2020). The resulting gene expression profiles were visualized as a heatmap *via* TBtools software (Chen et al., 2020a).

The expression profiles of the *KoWRKY* genes that responded to chilling stress were obtained from the NCBI transcriptomic database (Accession number: PRJNA678025). The expression of the *KoWRKY* genes was visualized as a heatmap using TBtools software (Chen et al., 2020a). For the transcriptome analysis of *KoWRKYs* in response to chilling stress, thresholds of *p* < 0.05 and |log_2_ (fold-change) ≥ 1| were used to define differentially expressed genes (DEGs).

### Experimental Validation of *KoWRKY* Genes Expression Levels via qRT-PCR

Total RNA was extracted using RNASimple Total RNA Kit (Tiangen, Beijing, China) and cDNA was obtained using TIANScript cDNA kit (Tiangen, Beijing, China) according to the manufacturers’ instructions. Following total RNA extraction and cDNA synthesis, quantitative analysis was performed via real-time PCR in conjunction with SuperReal PreMix Plus (SYBR Green, Tiangen, China) on a CFX96 Touch Real-Time PCR Detection System (Bio–Rad, United States) according to the manufacturers’ instructions. The PCR program included an initial denaturation step at 95°C for 30 s, followed by 40 cycles of 95°C for 10 s, 58°C for 10 s, and 72°C for 30 s. The relative transcript levels of the candidate genes were calculated according to the 2^−ΔΔCT^ method (Livak and Schmittgen, 2001). All the data were generated from averages of three independent replicates, and statistical significance was determined by one-way ANOVA followed by Duncan’s multiple range tests. All the primers used are listed in Supplementary Table S1.

### Analysis of Coexpression Networks Between *KoWRKY* Gene and Potential Regulated Gene

Differentially expressed *KoWRKY* gene was used as analysis object. Pearson correlation coefficient (PCC) between *KoWRKY* gene and non-*KoWRKY* gene was calculated to determine the coexpressed gene pair. Only gene pair with |PCC| value greater than 0.95 (*p* < 0.05) was used to regard as the potential regulated gene. The network was visualized using Cytoscape software (version 3.6.1).

## Results

### Identification and Characterization of *KoWRKY* Genes

To identify the WRKY-encoding genes present in the genome of *K. obovata*, the hidden Markov model (HMM) profile PF03106 from the Pfam database was used as a query for an HMM search against the genome, and a local BLASTP search was performed for which all 72 *A. thaliana* WRKY protein sequences were used in the query. In total, 64 potentially WRKY-encoding genes were identified and annotated. The 64 genes were named as KoWRKY1-KoWRKY64 according to the order of their gene ID, and the location on the chromosome, length of CDS, and the MW, pI and subcellular localization of their encoded proteins were shown in Table 1. Each chromosome contained *KoWRKY* members, of which chromosome 12 had the most, with 9 members; chromosome 18 had the fewest members, with only one. The CDS length ranged from 351 (*KoWRKY23*) to 2,181 bp (*KoWRKY35*), with an average length of 1,203 bp. The MW ranged from 12.99 kDa (KoWRKY23) to 78.60 kDa (KoWRKY35), with an average of 43.92 kDa. The theoretical pIs varied from 4.46 (KoWRKY33) to 10.26 (KoWRKY41), and the predicted subcellular localization results indicated that the 64 KoWRKYs were located in the nucleus (Table 1).

**TABLE 1:** Annotation of *Kandelia obovata* WRKY transcription factors.

Name	Gene ID	Group	Chromosome	Coding Sequence Length/bp	Protein Length/aa	Relative Molecular Weight/kDa	Theoretical Isoelectric Point (pI)	Subcellular Localization
KoWRKY1	GWHGACBH000028	III	1	1,140	379	41.67	5.61	Nucleus
KoWRKY2	GWHGACBH000887	IIe	3	966	321	34.7	9.19	Nucleus
KoWRKY3	GWHGACBH001429	I	3	1782	593	65.33	6.42	Nucleus
KoWRKY4	GWHGACBH001604	IIc	4	690	229	26.08	9.28	Nucleus
KoWRKY5	GWHGACBH002143	I	4	1830	609	66.94	6.41	Nucleus
KoWRKY6	GWHGACBH002442	IIc	14	909	302	32.75	5.67	Nucleus
KoWRKY7	GWHGACBH002459	I	14	1,539	512	55.56	8.48	Nucleus
KoWRKY8	GWHGACBH002730	I	7	2,124	707	76.97	5.72	Nucleus
KoWRKY9	GWHGACBH002784	I	7	1821	606	65.46	8.03	Nucleus
KoWRKY10	GWHGACBH002876	IIc	7	609	202	23.29	7.63	Nucleus
KoWRKY11	GWHGACBH003097	IIe	7	1,050	349	38.14	9.57	Nucleus
KoWRKY12	GWHGACBH004007	IIe	10	1,053	350	37.84	9.51	Nucleus
KoWRKY13	GWHGACBH004049	I	10	1,197	398	43.53	7.97	Nucleus
KoWRKY14	GWHGACBH004052	IIa	10	1,056	351	39.03	8.85	Nucleus
KoWRKY15	GWHGACBH004948	IIe	5	978	325	37.09	10.06	Nucleus
KoWRKY16	GWHGACBH005530	III	5	930	309	34.98	7.7	Nucleus
KoWRKY17	GWHGACBH005786	I	5	1,395	464	50.98	8.68	Nucleus
KoWRKY18	GWHGACBH005967	I	5	963	320	35.81	5.4	Nucleus
KoWRKY19	GWHGACBH006079	IIc	5	894	297	32.95	5.05	Nucleus
KoWRKY20	GWHGACBH006691	I	13	1,425	474	51.38	8.43	Nucleus
KoWRKY21	GWHGACBH006702	IIc	13	927	308	33.18	6.46	Nucleus
KoWRKY22	GWHGACBH007120	IIe	2	510	169	18.8	8.39	Nucleus
KoWRKY23	GWHGACBH007304	IIc	2	351	116	12.99	9.35	Nucleus
KoWRKY24	GWHGACBH007535	IIc	2	1,035	344	38.06	7.13	Nucleus
KoWRKY25	GWHGACBH008207	I	2	1,539	512	55.58	8.1	Nucleus
KoWRKY26	GWHGACBH008472	IId	9	1,272	423	46.08	5.43	Nucleus
KoWRKY27	GWHGACBH008891	IIb	9	1,650	549	58.8	6.87	Nucleus
KoWRKY28	GWHGACBH008978	IIc	9	531	176	20.19	9.67	Nucleus
KoWRKY29	GWHGACBH009250	I	12	1,431	476	51.6	5.75	Nucleus
KoWRKY30	GWHGACBH009302	IIb	12	1926	641	69.08	6	Nucleus
KoWRKY31	GWHGACBH009474	IIc	12	966	321	35.77	8.12	Nucleus
KoWRKY32	GWHGACBH009540	IIc	12	894	297	32.66	6.67	Nucleus
KoWRKY33	GWHGACBH009550	IId	12	858	285	32.38	4.46	Nucleus
KoWRKY34	GWHGACBH009725	IIe	12	1,065	354	39.93	9.69	Nucleus
KoWRKY35	GWHGACBH010316	I	6	2,181	726	78.6	6.08	Nucleus
KoWRKY36	GWHGACBH010385	I	6	1,680	559	60.62	6.4	Nucleus
KoWRKY37	GWHGACBH010420	IIb	6	1,047	348	37.78	8.75	Nucleus
KoWRKY38	GWHGACBH010479	IIc	6	618	205	23.46	7.64	Nucleus
KoWRKY39	GWHGACBH010849	IIe	6	1,143	380	41.33	9.61	Nucleus
KoWRKY40	GWHGACBH011057	I	1	2,100	699	75.95	5.76	Nucleus
KoWRKY41	GWHGACBH011286	IIe	1	1,389	462	50.67	10.26	Nucleus
KoWRKY42	GWHGACBH012351	IId	11	1,308	435	47.38	5.33	Nucleus
KoWRKY43	GWHGACBH012792	IId	4	942	313	34.2	9.74	Nucleus
KoWRKY44	GWHGACBH013356	IIe	8	702	233	26.4	9.81	Nucleus
KoWRKY45	GWHGACBH013438	III	8	1,035	344	38.33	5.57	Nucleus
KoWRKY46	GWHGACBH013593	IIc	8	936	311	34.3	6.55	Nucleus
KoWRKY47	GWHGACBH013762	IIc	8	960	319	35.39	8.05	Nucleus
KoWRKY48	GWHGACBH013975	IIb	8	1710	569	62.07	6.06	Nucleus
KoWRKY49	GWHGACBH014415	IId	15	984	327	36	5.38	Nucleus
KoWRKY50	GWHGACBH014450	III	15	1,026	341	38.33	6.09	Nucleus
KoWRKY51	GWHGACBH015228	IIb	16	1788	595	64.24	7.15	Nucleus
KoWRKY52	GWHGACBH015626	I	16	1770	589	64.55	6.79	Nucleus
KoWRKY53	GWHGACBH016027	IId	15	1,335	444	48.49	5.15	Nucleus
KoWRKY54	GWHGACBH016181	IIc	15	867	288	32.27	5.92	Nucleus
KoWRKY55	GWHGACBH016469	I	1	1716	571	62.72	6.58	Nucleus
KoWRKY56	GWHGACBH016668	I	1	1,416	471	51.77	8.44	Nucleus
KoWRKY57	GWHGACBH016971	III	1	945	314	35.35	6.08	Nucleus
KoWRKY58	GWHGACBH017668	IIb	12	1,548	515	55.33	8.9	Nucleus
KoWRKY59	GWHGACBH017748	III	12	1,059	352	39.06	5.9	Nucleus
KoWRKY60	GWHGACBH017774	IId	12	1,056	351	37.97	6.1	Nucleus
KoWRKY61	GWHGACBH018126	I	17	1,656	551	61	7.25	Nucleus
KoWRKY62	GWHGACBH018261	IId	17	750	249	27.56	7.05	Nucleus
KoWRKY63	GWHGACBH018617	III	11	1,029	342	38.52	5.32	Nucleus
KoWRKY64	GWHGACBH019047	IIa	18	975	324	35.83	7.99	Nucleus

### Chromosomal Localization, Exon/Intron Structure, and *Cis*-Elements in the Promoters of *KoWRKY* Genes

**FIGURE 1:**
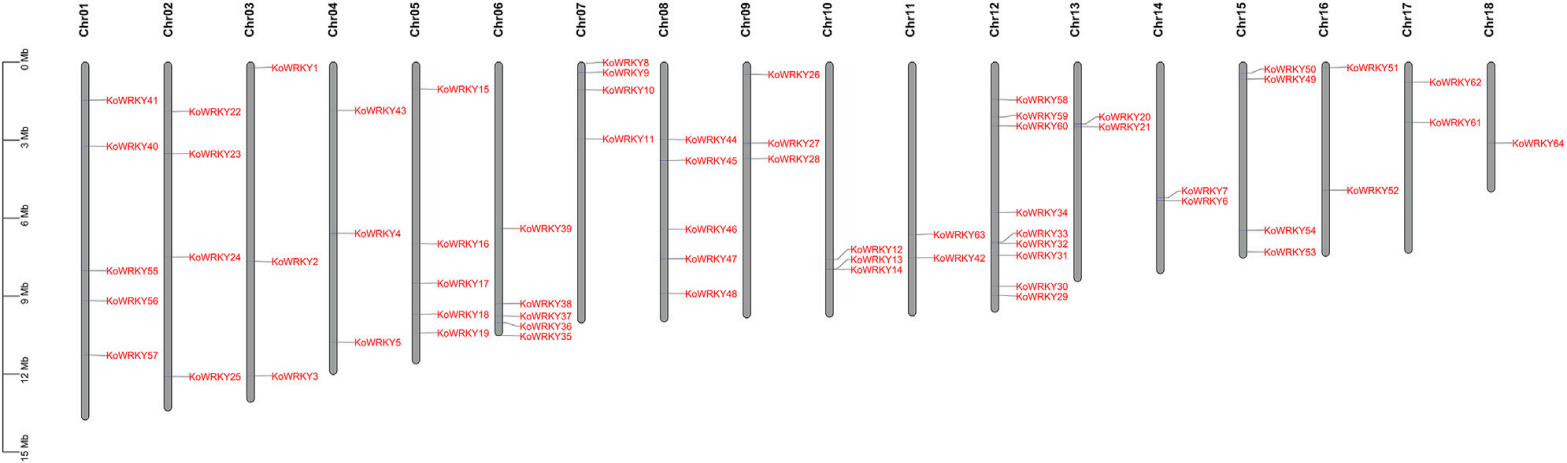
Chromosomal distribution of KoWRKY genes. The position of each *KoWRKY* gene on the chromosomes can be determined by the left scale.

To determine the distribution of the *KoWRKY* genes across the genome, all 64 identified KoWRKY mRNAs/open reading frames (ORFs) were mapped onto their corresponding chromosome via BLAST searches against the released *K. obovata* genome sequence. As shown in Figure 1, the *KoWRKY* genes were unevenly distributed across the 18 chromosomes, and the numbers on each chromosome were not related to chromosome length. Chromosome 12 had 9 *WRKY* genes (the majority), including one member from each of groups I, IIe and III of the *KoWRKY* gene family and 2 members from groups IIb, IIc, and IId, followed by 5 members on chromosomes 1, 5, 6, and 8; however, chromosome 18 contained only one *WRKY* gene (*KoWRKY* 64, in subgroup IIa).

**FIGURE 2:**
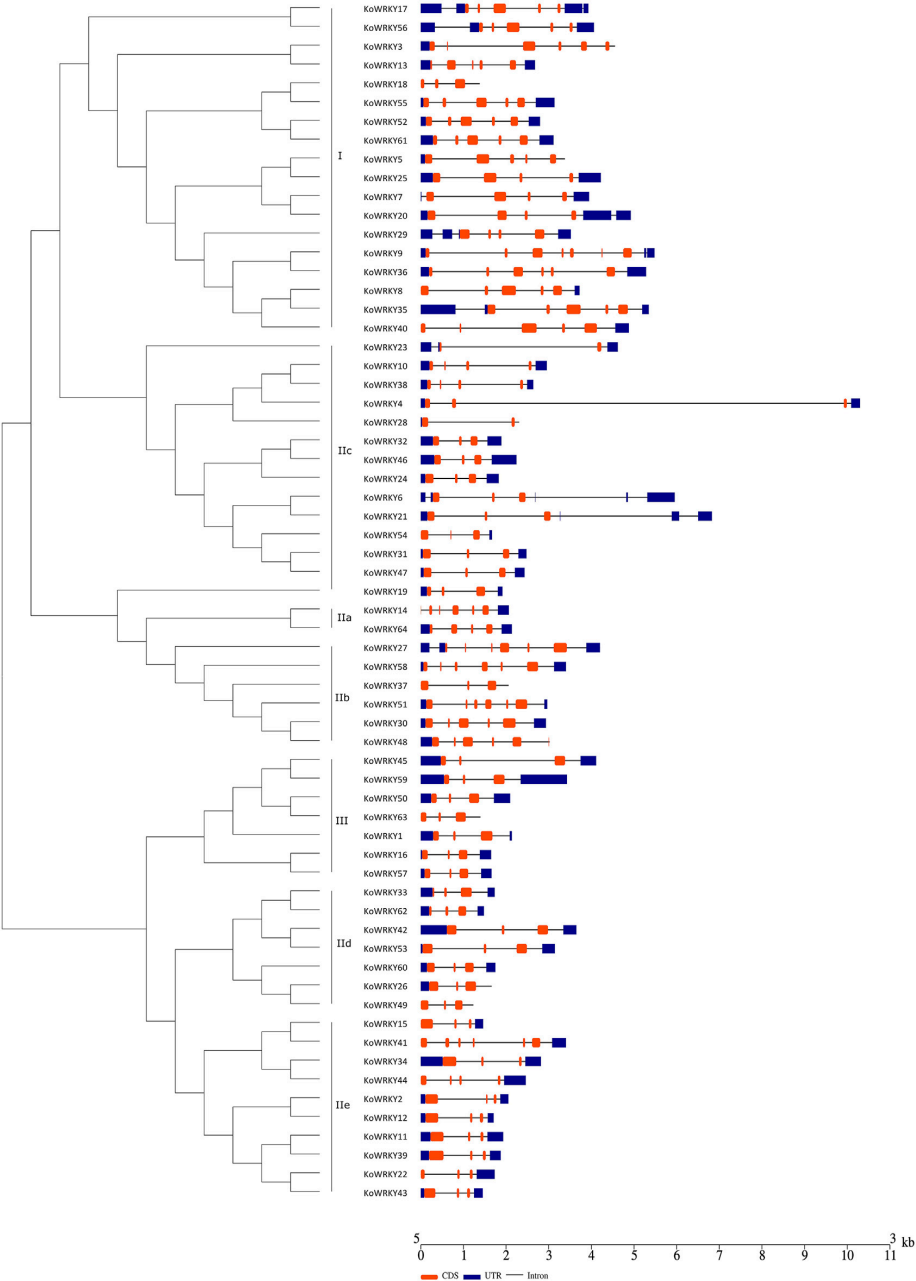
Gene structure analysis of *KoWRKY* genes from *Kandelia obovata*. Exon–intron structure analyses of the *KoWRKY* genes were performed *via* the Gene Structure Display Server (GSDS) online tools. The lengths of the exons and introns of each *KoWRKY* gene are shown proportional to each other. The introns are represented by black lines, and the exons and UTRs are represented by brown and blue boxes, respectively. A scale of gene length is given at the bottom. CDS, coding DNA sequence; UTR, untranslated region.

To investigate the structural diversity of the *KoWRKY* genes, the localization of intron–exon interactions was analyzed. It was found that the number of introns in the *KoWRKY* genes ranged from 1 (*KoWRKY23* and *KoWRKY28*) to 6 (*KoWRKY9*) in *K. obovata.* A total of 34 (53.13%) *KoWRKY* genes had 2 introns, followed by 8 (12.5%), 11 (17.19%), and 8 (12.5%) genes that contained 3, 4, and 5 introns, respectively (Figure 2). Most *KoWRKY* genes in group I had 3 to 5 introns, except *KoWRKY18* and *KoWRKY9*, which had 2 and 6 introns, respectively. The number of introns in the *KoWRKY* genes in group II widely varied, ranging from 1 to 5. However, all 7 *KoWRKY* genes in group III had 2 introns (Figure 2).

To analyze the functional diversification of the *WRKY* members, the sequences of the 2-kb upstream promoter regions of the *KoWRKY* genes were retrieved and analyzed. A number of *cis*-elements, including 9 elements related to plant development and 17 motifs related to stress responses, were analyzed, and the 26 elements are represented in Supplementary Table S2. The *cis*-elements related to plant growth and development include light-responsive elements (Box4, G-box, Sp1, ACE, and GT1 motifs), meristem-specific activation elements (CCGTCC-boxes), meristem expression-specific elements (CAT-boxes), endosperm expression-specific elements (GCN4 motif), and circadian control-related elements (circadians). *Cis*-elements related to stress responses include eight hormone-responsive elements (EREs, ABREs, CGTCA motifs, TGACG motifs, GARE motifs, TATC-boxes, P-boxes, TCA elements, and SAREs), low-temperature–responsive elements (LTRs and DRE cores), wound-responsive elements (WUN motifs), auxin response elements (TGAs and AuxRR cores), anaerobic induction elements (AREs), As shown in Supplementary Table S2, each *KoWRKY* gene contained more than one *cis*-element in its promoter region, and most *KoWRKY* genes contained Box4s, G-boxes, EREs, ABREs, TGACG and ARE motifs. Our analysis suggested that *KoWRKY* genes play an important role during development and stress responses.

### Gene Expansion, Selective Pressure and Synteny Analysis of *KoWRKY* Genes

To elucidate the mechanism underlying *KoWRKY* gene family expansion in *K. obovata*, BLASTP and MCScanX were employed to identify gene duplication patterns. The results showed that among the 64 *KoWRKY* genes, 49 segmentally duplicated genes formed 34 pairs (Supplementary Table S3); however, no tandem duplications were detected for *KoWRKY* genes, implying that segmental duplication was the major driving force for the expansion of *KoWRKY* genes.

To examine the selective pressure of *KoWRKY* genes, the Ka/Ks ratios of the duplicated gene pairs were calculated. Of the 35 paralogous *KoWRKY* gene pairs, the Ka values ranged from 0.05 to 0.54, the Ks values varied from 0.27 to 3.02, and all duplicated *KoWRKYs* had a Ka/Ks < 1, ranging from 0.12 to 0.45 (Supplementary Table S3). Estimated by a universal substitution rate of 6.1 × 10^–9^ mutations per site per year, duplications of KoWRKYs may have occurred at two time points, approximately 22.3–40.7 MYA and 194.6–247.8 MYA (Supplementary Table S3). Previous studies suggested that a Ka/Ks < 1, a Ka/Ks = 1, and a Ka/Ks > 1 indicate purifying selection, neutral evolution, and positive selection, respectively (Nan and Gao, 2019). In this study, none of the Ka/Ks ratios of repeated *KoWRKY* gene pairs in *K. obovata* were greater than 1, which indicates that they underwent purifying selection.

**FIGURE 3:**
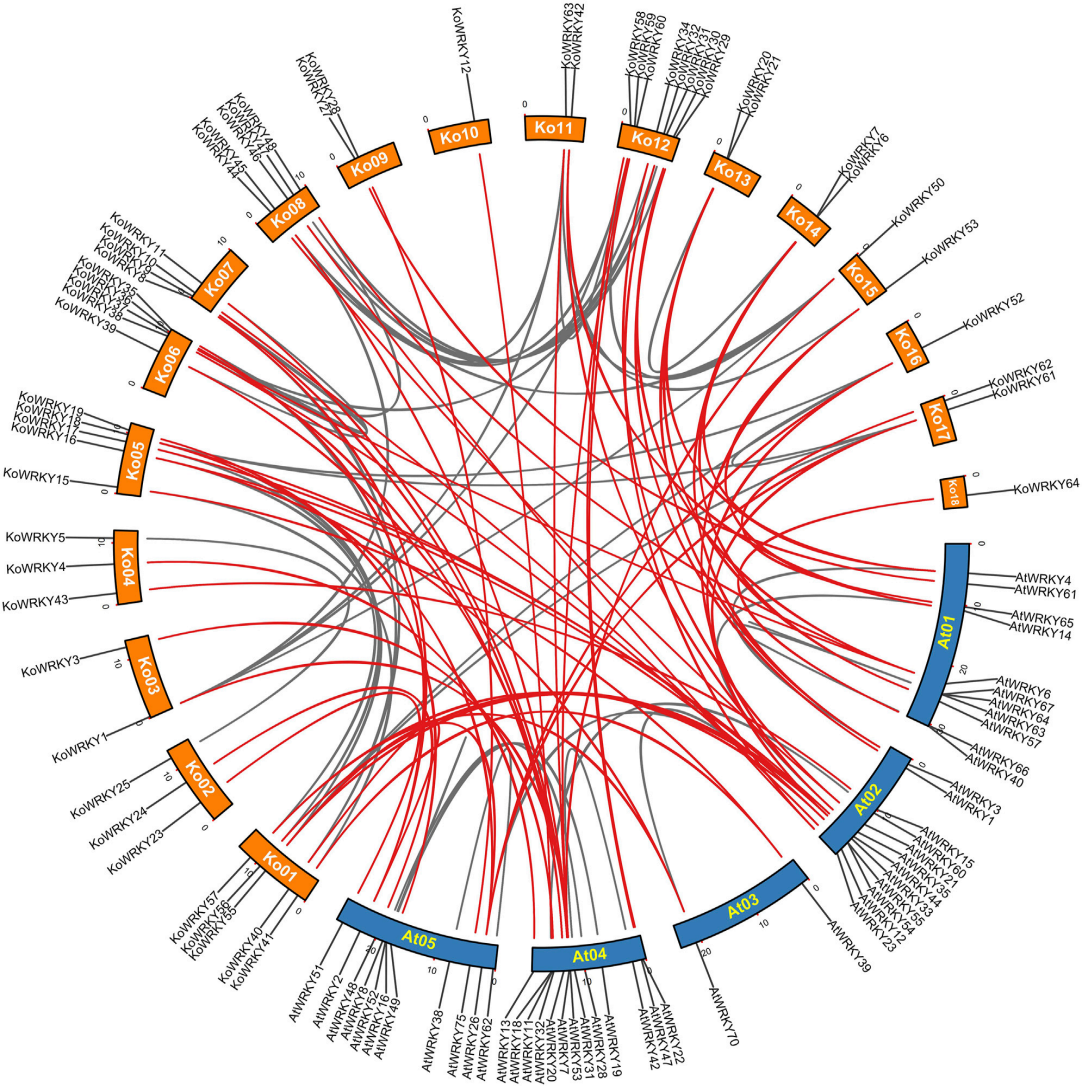
Synteny analysis between *WRKY* genes of *Kandelia obovata* and *Arabidopsis thaliana*, as performed by MCScanX. The chromosome numbers of *K. obovata* are Ko01–18, and the chromosome numbers of *A*. *thaliana* are At01–05. The red curves indicate synteny relationships between *K. obovata* and *A. thaliana WRKY* genes, and the gray curves indicate synteny between *WRKY* genes within *K. obovata* or *A*. *thaliana.*

To further explore the synteny relationships of *KoWRKY* genes with *A. thaliana,* Orthofinder was used to find the orthologous genes and TBtools was employed to construct the comparative synteny map (Figure 3). It showed that orthologous relationships between 50 *KoWRKY* genes and 32 *AtWRKY* genes were detected, and 59 orthologous *WRKY* gene pairs were identified based on these genes and the syntenic loci in *K. obovata* and *A. thaliana* chromosomes, suggesting that *WRKY* genes in both species had a similar origin and evolutionary process.

### Conserved Motifs, Sequence Alignment, and Phylogenetic Analysis of KoWRKY Proteins

**FIGURE 4:**
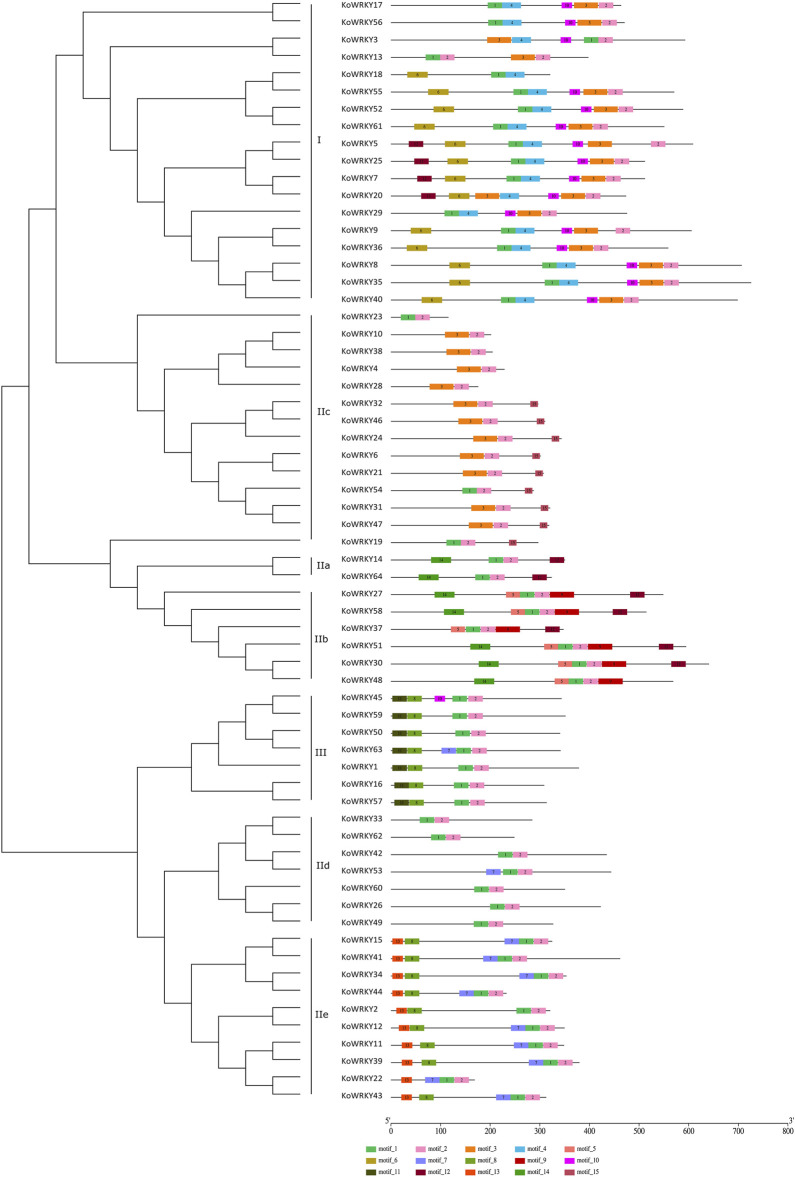
Conserved motifs of the KoWRKYs arranged according to their phylogenetic relationships. The ML tree shown was constructed from the amino acid sequences of KoWRKYs via ClustalX and MEGA 5, with 1,000 bootstrap replicates. The conserved motifs in the KoWRKYs were identified using MEME. In total, 15 motifs were identified and are shown in different colors. The motif locations are also indicated.

To gain insight into the functional regions of the KoWRKYs, the MEME program was used to identify the conserved motifs among the 64 KoWRKYs. A total of 15 conserved motifs were identified, namely, motifs 1–15 (Figure 4). These 15 conserved motifs are indicated with colored boxes according to their scale, with sizes ranging from 15 to 49 amino acid residues (Supplementary Figure S1). Among these individual motifs, motifs 1 and 3 were found to encode the conserved WRKY domain, and motifs 2 and 4 were found to encode the conserved zinc-finger structure. In addition to these conserved WRKY and zinc-finger motifs, other conserved motifs (motifs 5–15) were also predicted to be present within the KoWRKYs. Each KoWRKY protein had at least two conserved motifs, with the maximum number being seven, which was the case for several KoWRKYs. The distributions of the conserved motifs varied among the different KoWRKY groups. For example, group I KoWRKYs had seven motifs (motifs 1, 2, 3, 4, 6, 10, and 12), each containing one motif 1 and motif 3 (except KoWRKY18) and one zinc finger (motifs 2 and 4). Motifs 5, 6, 9, 11, 13, and 15 were present only in members of groups IIb, I, IIb, III, IIe and IIc, respectively. Motif 8 was present in members of group III and subgroup IIe, and motif 14 was present in subgroup IIa and IIb members. On the whole, the analysis of KoWRKY motifs showed that the KoWRKY members of every group or subgroup had similar motif compositions that corresponded to the clustering results generated by the phylogenetic tree analysis.

On the basis of the multiple alignment results for *K. obovata* WRKY protein sequences, the 64 WRKYs could be categorized into three groups (I, II and III) (Supplementary Figure S2). Most members of group I contained two conserved domains, WRKYGQK and a CX_4_CX_22-23_HXH zinc-finger motif in the N-terminal or a CX_4_CX_23_HXH motif in the C-terminal region. However, in KoWRKY18, the WRKYGQK sequence was replaced by WRKYGEK in the C-terminal region, and KoWRKY18 lacked a C-terminal WRKY domain. The 39 KoWRKYs in group II had a conserved heptapeptide WRKYGQK sequence (only KoWRKY23 contained a WRKYGEK sequence) and a CX_4_–_5_CX_23_HX_1_H zinc-finger domain, while 7 KoWRKYs in group III had a conserved WRKYGQK sequence and a CX_7_CX_23_HX_1_C zinc-finger domain.

**FIGURE 5:**
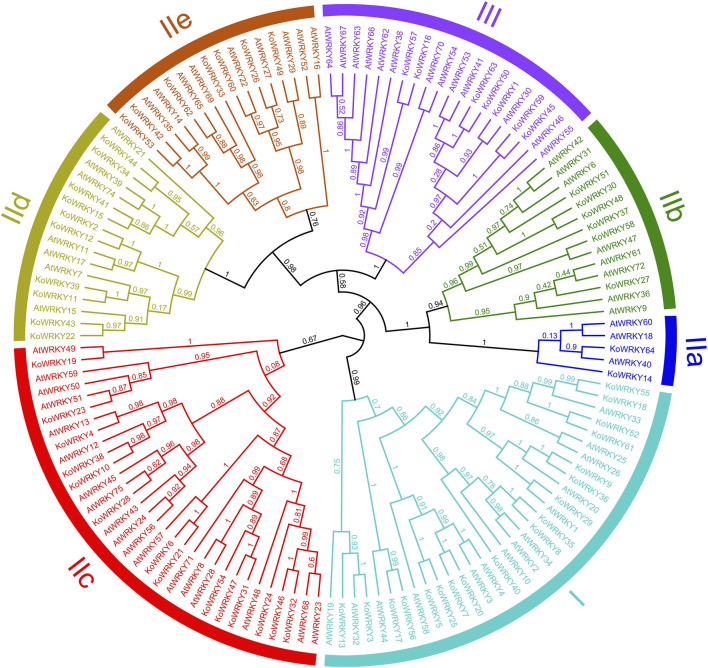
Phylogenetic relationships among WRKYs identified in *Kandelia obovata* and *Arabidopsis thaliana*. In total, 64 KoWRKY and 72 AtWRKY protein sequences were used to construct the phylogenetic tree using MEGA 7 and maximum likelihood (ML) method analysis (1,000 replicates). The *K. obovata* and *A. thaliana* genes are indicated at the ends of the branches. Subgroups I, IIa, IIb, IIc, IId, IIe, and III were named according to the results for *A. thaliana*. The colored regions indicate different subfamilies.

To investigate the evolutionary relationships between KoWRKYs and WRKYs from *A. thaliana*, an unrooted maximum-likelihood phylogenetic tree based on multiple alignments of the predicted amino acid sequences of the WRKY domains from *K. obovata* and *A. thaliana* was constructed. According to the tree (Figure 5), the KoWRKYs could be classified into three primary groups (groups I, II and III), with 18 KoWRKYs in group I, 39 KoWRKYs in group II, and 7 KoWRKYs in group III. Moreover, the KoWRKYs in group II could be further classified into five subgroups (groups IIa, IIb, IIc, IId and IIe) containing 2, 6, 14, 10, and 7 members, respectively. Notably, among all these groups and subgroups, most KoWRKYs were the members of subgroup IIc, the case of which is similar to AtWRKYs.

### Potential Target Genes of KoWRKY Proteins Were Enriched Into 11 Significant Pathways by KEGG Analysis

**FIGURE 6:**
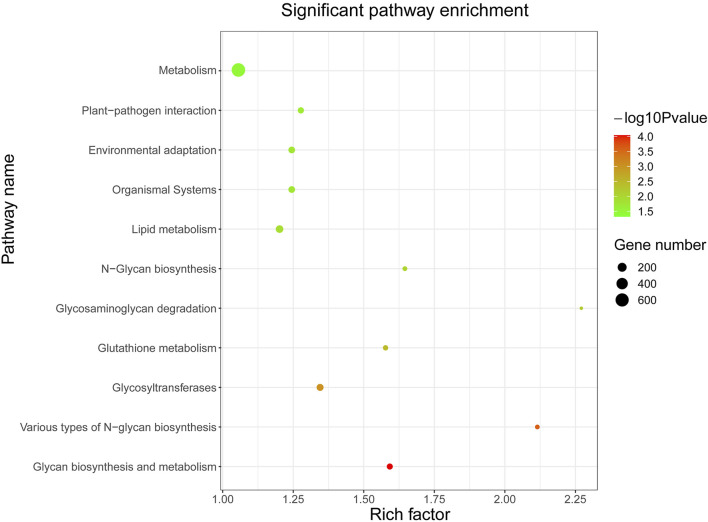
KEGG pathway enrichment analysis of predicted putative target genes of KoWRKYs in *K. obovata*. The coloring of the q-values represents the significance of the matched gene ratio; the circle size represents the target gene number.

In order to lay the foundation for the functional analysis of KoWRKY proteins, their potential target genes were subjected to KEGG enrichment analysis. KEGG pathway enrichment analysis showed that the potential target genes of KoWRKY proteins were significantly (*p* < 0.05) involved in 11 pathways (Figure 6). The three pathways of metabolism, lipid metabolism, and glycosyltransferases had the most enriched genes, which were 637, 113, and 86, respectively. In particular, 73 target genes were involved in environmental adaptation pathways (Supplementary Table S4), which may play an important role in how *KoWRKY* genes regulate the adaptation process of *K. obovata* to the environment.

### Expression Profiles of *KoWRKY* Genes in Different Tissues and in Response to Chilling Stress

**FIGURE 7:**
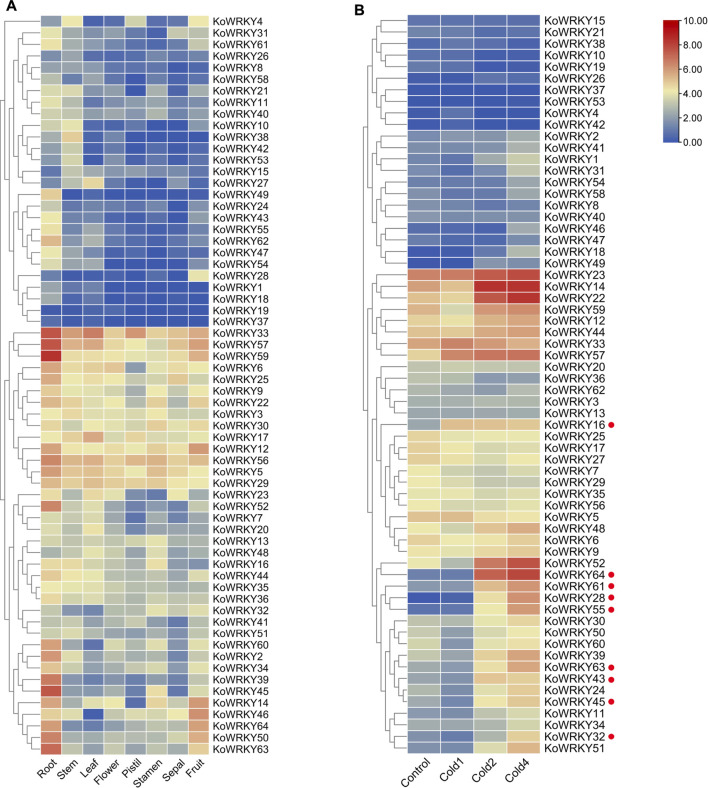
Expression of *KoWRKY* genes in *Kandelia obovata*. **(A)** The transcript levels of the *KoWRKY* genes in 8 tissues of *K. obovata* were investigated based on publicly available transcriptomic data. The color scale shows increasing expression levels from blue to red. **(B)** The transcript levels of *KoWRKY* genes in response to cold stress were investigated based on publicly available transcriptomic data. Cold1, first cold treatment; Cold2, second cold treatment; Cold4, fourth cold treatment. The genes whose expression increased more than two-fold after cold treatment are labeled with red dots.

The expression profiles of all 64 *KoWRKY* genes were investigated using a standard transcriptome analysis procedure based on publicly available transcriptomic data of different tissues of *K. obovata*, including root, stem, leaf, flower, pistil, stamen, sepal, and fruit tissues. Among the 64 *KoWRKYs*, 50 were expressed in samples above (TPM > 0). Some *KoWRKY* genes showed preferential expression across all the tissues tested. *KoWRKY* genes, especially *KoWRKY33*, *KoWRKY39*, *KoWRKY45*, *KoWRKY50*, *KoWRKY52*, *KoWRKY56*, *KoWRKY57*, *KoWRKY59* and *KoWRKY63*, were most highly expressed in the roots. Several members, such as *KoWRKY2*, *KoWRKY6*, *KoWRKY9*, *KoWRKY21*, *KoWRKY39*, *KoWRKY50*, *KoWRKY54*, *KoWRKY60*, and *KoWRKY62* presented higher expression levels in the fruits than in the other tissues. Among all the tissues, the fewest *KoWRKY* members were expressed the most in the sepals. In addition, *KoWRKY19* and *KoWRKY37* were barely expressed in any of the tissues tested (Figure 7A).

To investigate the potential functions of *KoWRKYs* in response to chilling stress, the expression of *KoWRKY*s after chilling treatment based on publicly available transcriptomic data was analyzed. The results showed that the expression levels of most *KoWRKY* genes, especially *KoWRKY16*, *KoWRKY28*, *KoWRKY32*, *KoWRKY43*, *KoWRKY45*, *KoWRKY55*, *KoWRKY61*, *KoWRKY63*, and *KoWRKY64*, were upregulated after chilling treatment, the expression of which increased more than two-fold. However, the expression levels of *KoWRKY37*, *KoWRKY42* and *KoWRKY53* were not different from those of the control after the first, second and fourth cold treatments (Figure 7B).

**FIGURE 8:**
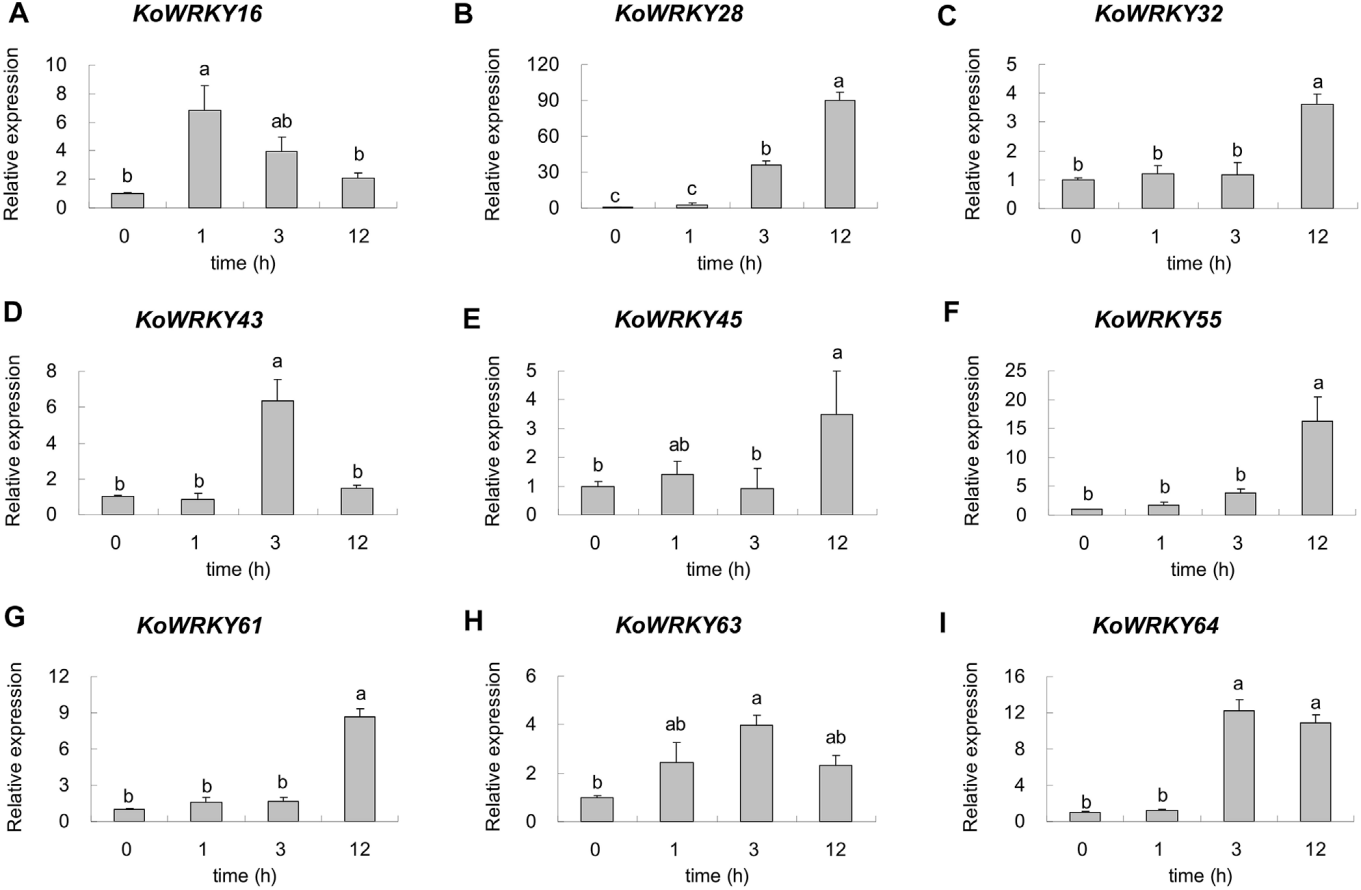
Expression profiles of nine selected *KoWRKY* genes in response to chilling stress. The relative expression levels of nine *KoWRKY* genes were measured in plants subjected to 4°C for 0, 1, 3, and 12 h. The transcript levels of the selected genes were assessed *via* qRT–PCR and normalized to 18S rRNA levels. The error bars represent the standard errors. The values with the same letter are not significantly different according to Duncan’s multiple range test (*p* < 0.05, *n* = 3). Panels **(A–I)** represented the expression level of gene *KoWRKY16, KoWRKY28, KoWRKY32, KoWRKY43, KoWRKY45, KoWRKY55, KoWRKY61, KoWRKY63*, and *KoWRKY64*, respectively.

To confirm the candidate *KoWRKY* genes that are important for cold tolerance, 9 DEGs were selected, and their expression levels were quantified via qRT–PCR. As shown in Figure 8, the expression of all the selected *KoWRKY* genes was upregulated under 4°C. Under low-temperature stress, the expression of *KoWRKY16* increased sharply but then decreased, peaking after 1 h, the level of which was significantly higher than that of the control. The expression of *KoWRKY43*, *KoWRKY63* and *KoWRKY64* peaked at 3 h after 4°C treatment; however, the expression of *KoWRKY28*, *KoWRKY32*, *KoWRKY45*, *KoWRKY55* and *KoWRKY61* peaked at 12 h. The largest increase in the expression level (approximately 90-fold) was detected for KoWRKY28 after 12 h of chilling treatment.

### 315 Significantly Correlated Coexpressed Pairs of *KoWRKYs* and mRNAs Were Identified

**FIGURE 9:**
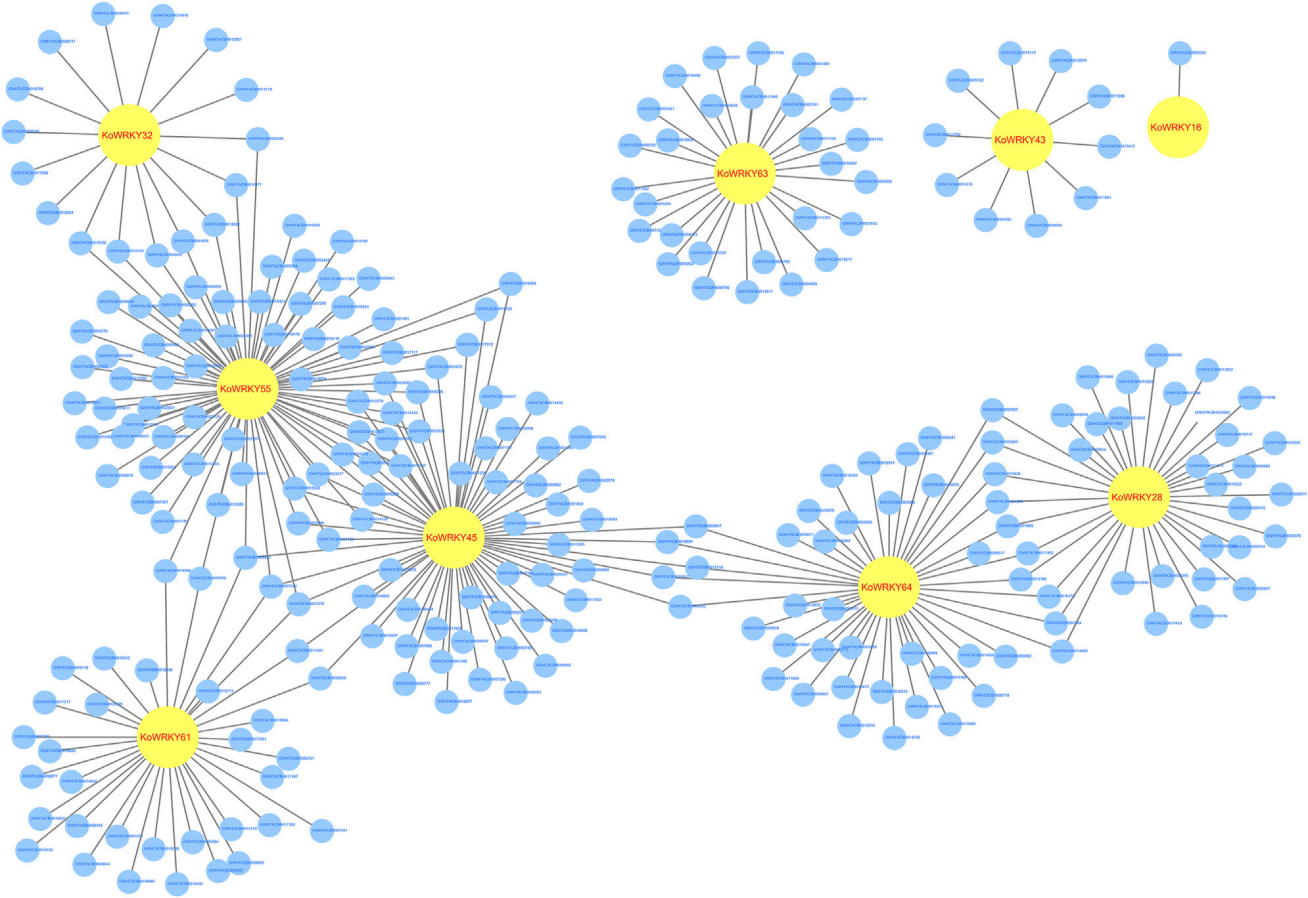
Coexpression network of *KoWRKY* genes with other genes in *Kandelia obovata* under cold stress. The coexpression network was established between the 9 *KoWRKYs* and 263 significantly expressed mRNAs whose Spearman correlation coefficients were equal to or greater than 0.95. Within this coexpression network, all 315 pairs were positive. The yellow circles represent *KoWRKY* genes, while the blue circles represent the coexpressed genes.

Genome-wide gene expression profiling of *KoWRKY16*, *KoWRKY28*, *KoWRKY32*, *KoWRKY43*, *KoWRKY45*, *KoWRKY55*, *KoWRKY61*, *KoWRKY63*, and *KoWRKY64* and mRNAs from leaves of plants subjected to chilling stress was conducted to identify genes coexpressed with *KoWRKYs*. The potential target mRNAs were predicted via Pearson correlation test for the 9 above mentioned *KoWRKY* genes whose expression increased more than two-fold after chilling treatment. The results showed that 263 significantly expressed mRNAs were correlated (PCC >0.95, *p* < 0.001) with 9 *KoWRKYs*, and for all 315 coexpressed pairs, each *KoWRKY* and mRNA was positively correlated (Figure 9, Supplementary Table S5). Taken together, the results indicated that the *KoWRKYs* might positively regulate the response of these putative genes in chilling stress.

## Discussion

*WRKY* gene family members are types of TFs that have long been indicated to regulate multiple physiological processes in plants. Since Ishiguro and Nakamura (1994) cloned the first WRKY gene (SPF1) from sweet potato, it has been cloned from the roots, leaves, inflorescences, seeds, and microstructures of various plant species, such as *A. thaliana*, *Oryza sativa*, *Zea mays*, and *Medicago truncatula*. With the continuous completion of genome sequencing of different species, an increasing number of *WRKY* genes have been identified, and their biological functions have also been extensively explored. Specifically, WRKY TFs have been shown to regulate a variety of processes in response to biotic and abiotic stress in plants (Eulgem et al., 2000). However, studies of the *WRKY* gene family of *K. obovata* have not yet been reported. In the present study, a total of 64 *WRKY* gene members in *K. obovata* were identified via bioinformatics methods to further analyze their structure, function and expression, aiming to provide some reference for follow-up studies on *KoWRKY* gene function and regulatory mechanisms.

According to the number of conserved WRKY regions and the patterns of zinc-finger motifs, all of the *WRKY* genes can be classified into three groups: groups I, II, and III (Eulgem et al., 2000). Group I members have two conserved WRKY domains and a C_2_H_2_ zinc-finger motif; group II members have only one conserved WRKY domain and the same zinc-finger motif as group I members have; and group III members have one conserved WRKY domain and a C_2_HC zinc-finger motif (Rushton et al., 2010). In the present study, the KoWRKYs were categorized into three groups based on the conserved domains of the proteins, and the results were consistent with previous findings. Almost all of the KoWRKYs shared the highly conserved WRKYGQK domain; however, variants of the WRKYGEK and WRKYGKK domains could still be found in KoWRKY13 and KoWRKY23, respectively. Such variations in the WRKYGQK conserved motif have also been reported in many other species. For example, six OnWRKYs (OnWRKY18, OnWRKY46, OnWRKY52, OnWRKY55, OnWRKY84, and OnWRKY114) in *O. nivara* had a WRKYGEK domain instead of the WRKYGQK conserved domain (Xu et al., 2016), and four VvWRKYs (VvWRKY8, VvWRKY13, VvWRKY14, and VvWRKY24) from *V. vinifera* contained the variant WRKYGKK rather than the WRKYGQK motif (Guo et al., 2014). Previous studies demonstrated that the WRKYGQK domain can bind to the core W-box *cis*-element motif (C/T)TGAC(C/T) to activate the expression of downstream genes (Eulgem et al., 2000). The variation in or loss of the conserved WRKYGQK domain might affect the specificity of binding to *cis*-elements (Ciolkowski et al., 2008). For example, the AtWRKY59 protein in *A. thaliana* could not bind to TTGAC (a W-box) because its WRKYGKK motif was replaced with WRKYGQK (Dong et al., 2003). Furthermore, in *Nicotiana tabacum* NtWRKY12 with a WRKYGKK domain could bind to WK-box elements rather than W-box motifs (van Verk et al., 2008). Thus, further efforts are needed to experimentally prove the binding specificities of the KoWRKY13 and KoWRKY23 proteins with variants of the WRKYGQK motif.

In addition to having a variant of the highly conserved WRKYGQK domain, some WRKYs may lack conserved motifs. For instance, both AtWRKY10 (group I) in *A. thaliana* and CsWRKY42 in *Cucumis sativus* have only one WRKY domain (Wei et al., 2012; Chen et al., 2020b). The same result occurred in this paper, for KoWRKY18 (group I) lost its C-terminal WRKYGQK domain. It was reported that the C-terminal domain of WRKYs (group I) was sufficient for W-box element recognition, whereas the N-terminal WRKY domain alone failed to bind W-box element, but it may increase the overall binding affinity of the protein to DNA by making additional contacts with DNA or by interacting with other proteins (Eulgem et al., 1999; Maeo et al., 2001). Therefore, loss of the C-terminal WRKY domain might influence the recognition and binding of KoWRKY18 to target genes, and further study of the binding specificities and functions of the KoWRKY18 protein might be worthwhile.

It was found that the number of WRKY genes in different species is not positively related to the size of their genome. For instance, *A. thaliana* genome is only 125 Mb and contains 72 WRKY genes. *C. melo* and *O. sativa* ssp. *japonica* have similar genome sizes (450 and 466 Mb, respectively); however, the former contains 65 WRKY genes, and the latter contains 128 WRKY genes (http://planttfdb.gao-lab.org/family.php?fam=WRKY, 2022.2.14). In the present study, the *K. obovata* genome size was 180 Mb, but the genome was found to contain 64 members of the WRKY family. Although the number of *WRKY* genes is nearly the same, the genome size of *C. melo* is more than 2 times that of *K. obovata*. Recent studies have proposed that gene duplication is considered to be one of the primary driving forces in the expansion of gene families and genome evolution, and the major duplication patterns are tandem duplication and segmental duplication (Cannon et al., 2004). Tandem duplications have been reported to play major roles in the expansion of the *WRKY* family in Solanum tuberosum (Shi et al., 2017) and *Citrus sinensis* (Silva et al., 2017); however, segmental duplications seem to be more common than tandem duplications are in the expansion of the *WRKY* family, such as in *O. rufipogon* (Nan et al., 2020), *Cicer arietinum* (Waqas et al., 2019), and *Ananas comosus* (Xie et al., 2018). These findings are consistent with research in *Camelina sativa* (Song et al., 2020). Our results showed that 49 segmental duplication events were present in 64 *KoWRKY* genes; however, no tandem duplication was detected for any *KoWRKY* gene. These events revealed that segmental duplication was the major driving force for the expansion of *KoWRKY* genes. The Ks value is widely used to estimate the evolutionary history of segmental duplication events. It was reported that the Ks distributions for paralogous *K. obovata* genes exhibited two peaks, one at Ks = 0.38 and the other at Ks = 1.5–1.9 (Hu et al., 2020). These results are consistent with the results of the present study, in which the mean Ks value of the *KoWRKY* genes in the present paper exhibited two peak values, 0.39 and 2.56, which implied that *K. obovata* underwent two segmental duplications in recent years.

As important types of TFs, by acting as positive or negative regulators WRKYs, regulate the responses to biotic and abiotic stresses of plant species such as *A. thaliana*, *O. sativa* and *Glycine max* (Rushton et al., 2010). However, there is still no relevant research on *WRKY* genes in *K. obovata*. Studies have shown that the tissue-specific expression of *WRKY* genes exerts strong effects during plant growth and development by regulating the expression of gene involved in growth and differentiation (Li et al., 2014). In the present study, a large number of *KoWRKY* genes were found to be constitutively expressed in the roots of *K. obovata*, with many of them, such as *KoWRKY27*, *KoWRKY28*, *KoWRKY38*, *KoWRKY39*, and *KoWRKY49*, showing a tissue-specific expression pattern (Figure 7A), implying that *KoWRKYs* have vital functions in plant development and function differently in different tissues. *K. obovata*, a dominant mangrove species distributed along the southern coast of China, survives in harsh environments and experiences environmental stresses such as submergence, hypoxia, salinity, and even extremely low temperatures in winter (Fei et al., 2021; Nizam et al., 2022). Hypoxia (which can be caused by submergence or waterlogging) and salinity stresses affect the survival and growth of many plants, some of which have developed multiple strategies to cope with these stressful conditions during their evolution, including morphological changes and scavenging of reactive oxygen species (Tang et al., 2020). However, the most critical mechanism is based on gene regulation involving signaling cascades, in which responses to hypoxia or salinity signals are triggered. Recent studies have also demonstrated that *WRKY33*- and *WRKY12-*overexpressing *A. thaliana* showed enhanced resistance to hypoxia (Tang et al., 2020), and overexpression *of VvWRKY30* in *A. thaliana* increased resistance to salt stress at different stages of growth (Zhu et al., 2019). As such, many *KoWRKY* genes showed a high expression pattern in the roots, implying that these TF genes play vital roles in the adaptation of those plants to mudflat environments. Further research on *KoWRKY* function and the regulatory mechanisms should be conducted.

In the present study, RNA-seq revealed that the expression of some *KoWRKYs* in the leaves of *K. obovata* changed under chilling stress. Among these *KoWRKYs*, most were found to be upregulated in response to chilling stress, but some were downregulated. Similar results were also found in *Coffea canephora* (Dong et al., 2019) and *Prunus mume* (Bao et al., 2019), indicating that TF *KoWRKYs* might act as positive or negative regulators. In addition, nine putative candidate *KoWRKY* genes (*KoWRKY16*, *KoWRKY28*, *KoWRKY32*, *KoWRKY43*, *KoWRKY45*, *KoWRKY55*, *KoWRKY61*, *KoWRKY63*, and *KoWRKY64*) were upregulated more than twofold after low-temperature treatment. The expression profile generated by qRT–PCR in this work showed that the nine candidate *KoWRKY* genes were upregulated after treatment with 4°C (Figure 8), which in most cases coincides with the expression patterns obtained *via* RNA-seq. It has been reported that over-expression of *OsWRKY71* in rice enhanced the tolerance to chilling stress (Kim et al., 2016); moreover, over-expression of *BcWRKY46* has been shown to increase the chilling and freezing tolerance of tobacco (Wang F et al., 2012). Similar functions were also found in the study of genes *CsWRKY46* (Zhang et al., 2016) and *GmWRKY21* (Zhou et al., 2008). From the phylogenetic tree, it can be seen that *KoWRKY64* and *OsWRKY71*, *KoWRKY45* and *BcWRKY46* are very closely related (Supplementary Figure S3), which means that *KoWRKY64* and *KoWRKY45* may play an important role in coping with cold stress. The results suggest that these genes potentially are involved in the chilling resistance of *K. obovata*. Nonetheless, further experimental analyses should be carried out to elucidate the precise regulatory mechanism through which *KoWRKY* genes respond to chilling stress.

## Conclusion

In conclusion, 64 *KoWRKYs* were identified in the genome of *K. obovata*, and they were unevenly distributed across all 18 chromosomes. The evolution, gene structure and *cis*-elements in the promoter regions of the *KoWRKYs* were also analyzed. Some *KoWRKYs* were highly expressed in specific tissues, and 9 *KoWRKYs* in the leaves were significantly induced in response to chilling stress. These genes represent candidates for future functional analysis of *K. obovata* in response to low temperature. Our results provide a basis for further analysis of *KoWRKY* genes to determine their function and elucidate the molecular mechanisms underlying the response of *K. obovata* to chilling stress.

## Data Availability

The original contributions presented in the study are included in the article/Supplementary Materials, further inquiries can be directed to the corresponding author.

## Supplementary Material

Supplementary Figure S1 Details of 15 motifs of KoWRKYs.

Supplementary Figure S2 Multiple sequence alignment of the WRKY domain from KoWRKYs. Alignment was performed using DNAMAN. The conserved heptapeptide is WRKYGQK and the zinc-finger motif are C2H2 or C2HC. The color shade of the amino acid residues highlighted the homology level: dark blue = 100%, pink ≥75%, and cambridge blue ≥50%.

Supplementary Figure S3 Phylogenetic relationships among WRKYs identified with other species. In total, 64 KoWRKY, 72 AtWRKY, 1 BcWRKY46, 1 CsWRKY46. 1 GmWRKY21, and 1 OsWRKY71 protein sequences were used to construct the phylogenetic tree using MEGA 7 and maximum likelihood (ML) method analysis (1000 replicates). Subgroups I, IIa, IIb, IIc, IId, IIe, and III were named according to the results for *A. thaliana*. The colored regions indicate different subfamilies. The Genbank accession no. of BcWRKY46, CsWRKY46. GmWRKY21, and OsWRKY71 were ADM32893, ADU52524, ABC26913 and AAT84158, respectively. The two purple and blue and dots represented the closely related sequences.
