# Supplementary figures and images for: Genome-Wide Identification of WRKY Genes and Their Responses to Chilling Stress in Kandelia obovata
Source: Front Genet. 2022 Mar 31;13:875316. doi: 10.3389/fgene.2022.875316 (PMC9008847; doi:10.3389/fgene.2022.875316)

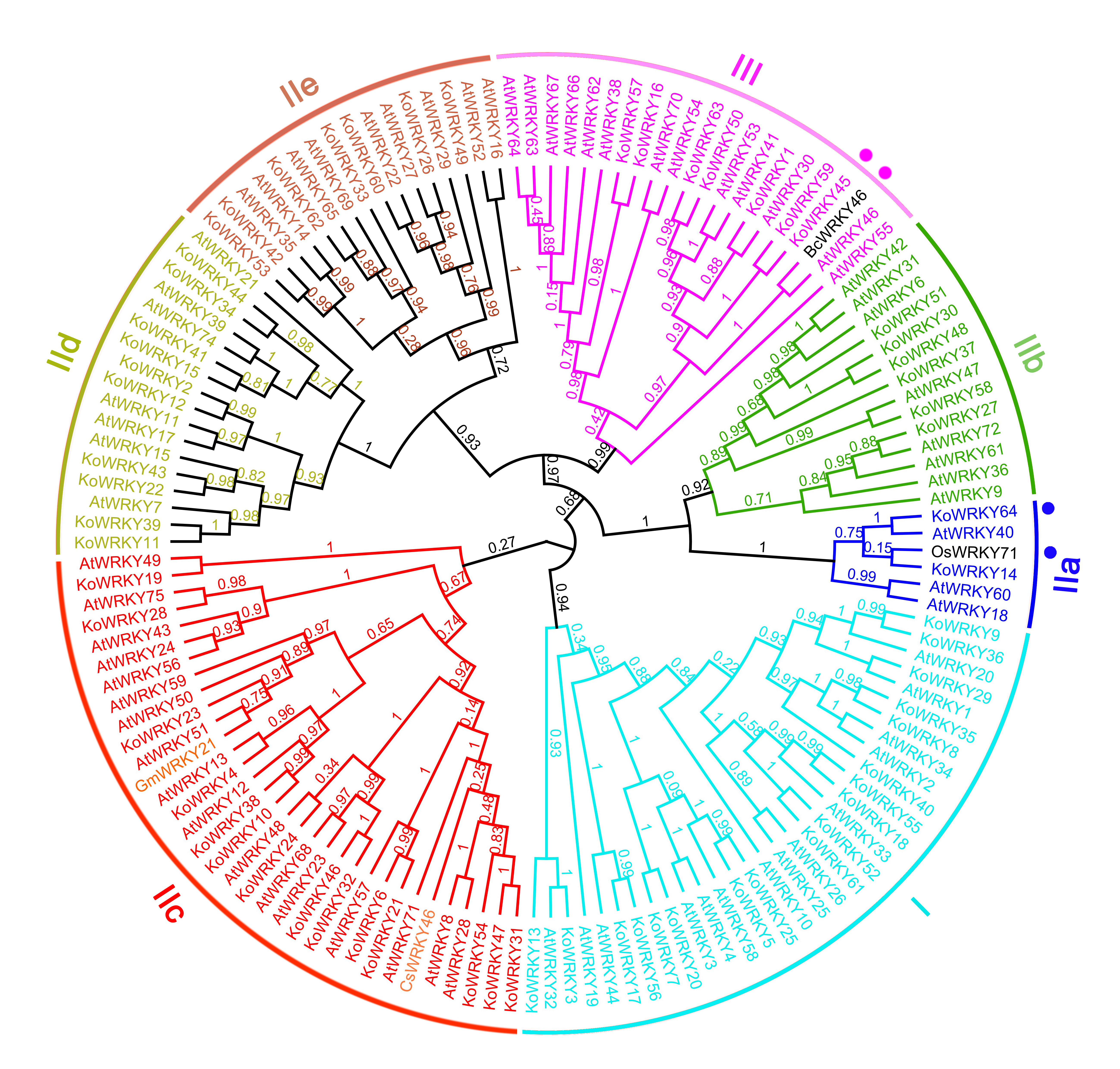

Supplement: Supplementary file 1 [file Image3.JPEG]

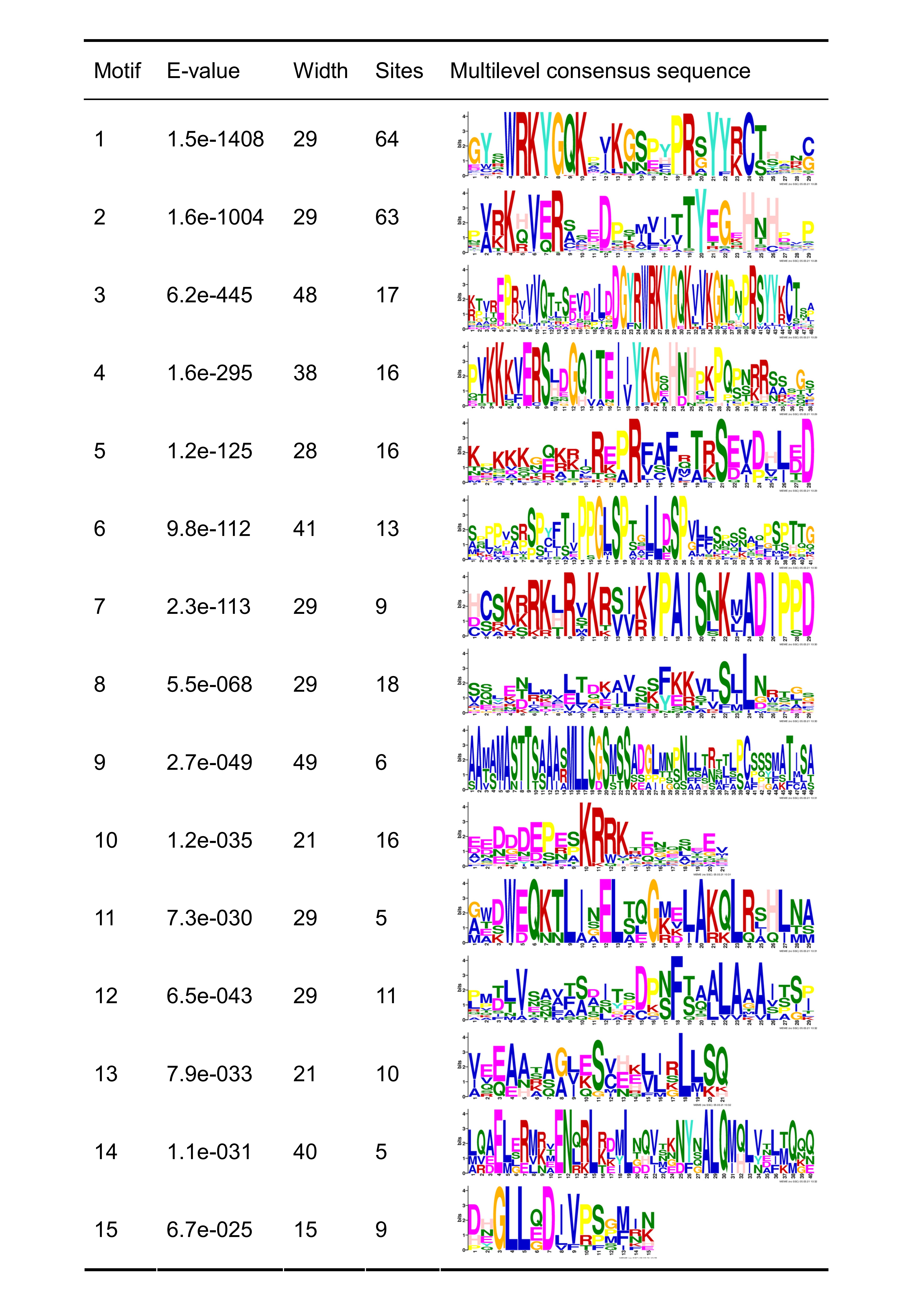

Supplement: Supplementary file 4 [file Image1.JPEG]

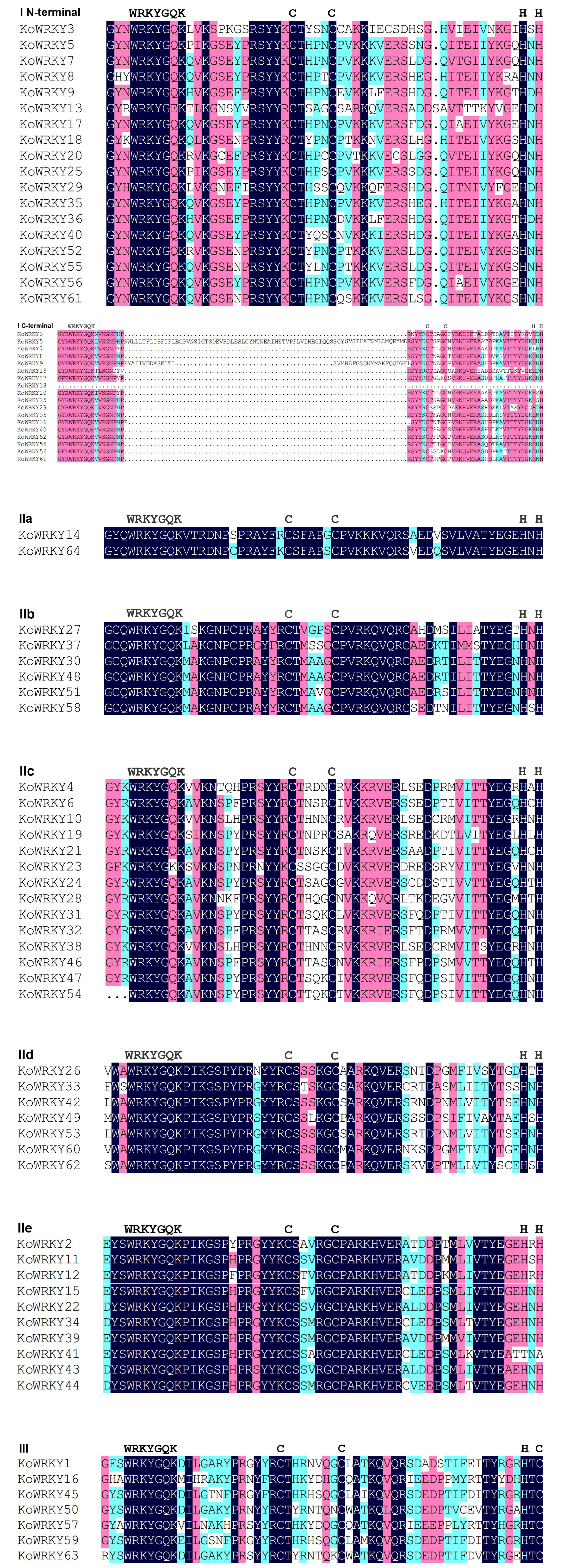

Supplement: Supplementary file 6 [file Image2.JPEG]
