# Supplementary material for: Genome-Wide Identification of WRKY Genes and Their Responses to Chilling Stress in Kandelia obovata
Source: Front Genet. 2022 Mar 31;13:875316. doi: 10.3389/fgene.2022.875316 (PMC9008847; doi:10.3389/fgene.2022.875316)
Supplement: Supplementary file 8 [file Table1.DOC]

Supplementary Table S1. Primer used for qRT-PCR analysis on the target sequences in *K.obovata*

| KoWRKY | Primer pairs | |
| --- | --- | --- |
|  | Forward primer (5'-3') | Reverse primer (5'-3') |
| KoWRKY16 | CGAACTGGCAACCCGACTTA | GAATCCTCGGACTTTCGGCT |
| KoWRKY28 | GCCCAGACTTGCTTTTCAGAC | TCCTTGATGCGTACACCGAT |
| KoWRKY32 | TTGGGTTTGGGATACGGTGG | AGGGGAGGGAACGATGTCTT |
| KoWRKY43 | CACCGATCCAGCAGATTCCC | ACAAGAAGGACGAAAGCGGA |
| KoWRKY45 | TAGGCGACCATGTGCATACC | GCACCGGAAAAGTCCAAACC |
| KoWRKY55 | AAGTGAGAACCCACGCAGTT | CTGAGGCTTCGGATGGTTGT |
| KoWRKY61 | TTCTGTTTCCAGCCACCGTC | GCAGCAATCTCAGTGGGGAA |
| KoWRKY63 | TCCTTCTGCCTACGGGTTTG | ATCGAGGCTTGGGAAATCGT |
| KoWRKY64 | AACCGTAGCCTGACACTTGG | GGCATCATTTCTGGGCTTGG |
| 18S rRNA | GGGGCTCGAAGACGATCAGA | TTAAGCCGCAGGCTCCACTC |
